# The three-minute appraisal of a randomized trial

**DOI:** 10.4103/0019-5413.80036

**Published:** 2011

**Authors:** Katelyn Godin, Mandeep Dhillon, Mohit Bhandari

**Affiliations:** McMaster University, Canada; 1 PGIMER, Chandigarh, India

## INTRODUCTION

A randomized controlled trial (RCT) involves the random allocation of subjects into different study groups, usually a control group and an experimental group that receives the treatment under analysis, which is either a medical treatment or a surgery. RCTs conducted with proper methodology represent the gold standard of medical research and their outcomes can contribute to improved clinical practices and procedures.^1^,^2^ However, RCTs are vulnerable to several factors that impact the validity of the study.^1^,^2^ This paper outlines specific criteria that can be used in the appraisal of an RCT. A practical example is included to better demonstrate an application of the appraisal process.

## KEY CRITERIA FOR CRITICAL APPRAISAL AND THEIR EXPLANATION

When appraising an RCT, there are many factors to consider that can be accounted for in three general questions^3^[Table 1]:

**Table 1:** Checklist for evaluating a randomized trial

Are the results of trial valid? Was the learning curve taken into consideration? Were the subjects randomized? The randomization concealed? Were subject aware of group allocation? Were surgeons and outcome assessors aware of group allocation? Were the experimental and control group similar in terms of prognostic factors? Were subjects stratified? Were subjects analyzed in the group they were initially randomized into at enrolment? Was follow-up complete? What are the results of the trial? How were the results of the trial being measured? How significant is the treatment effect? Are the results applicable to clinical practice? How similar is your patient to the subjects included in the study? Do the benefits of the treatment outweigh the potential risks and costs?

### Are the results of the trial valid? (3-minute checklist)

The study’s design must be critically evaluated to determine the trial’s validity. With surgical RCTs, the learning curve must be addressed.^4^ In such trials, the target outcomes of a novel surgery are compared to that of an existing surgery. Surgeons are less prone to make mistakes with a traditional, more familiar surgery, thus there may be bias against the novel surgery.^4^ This bias can be reduced by requiring the participating surgeons to practice the particular surgery or take a course on the procedure before the trial. The minimum number of procedures will vary depending upon the specific technique and complexity of the expertise required.

Randomization of subjects into control group and experimental groups is crucial for a study’s validity. When assignment is random, for example by a computer program, this bias is eliminated.^4^ Surgeons should not be able to “guess” or “determine” which treatment the next patient on the trial will receive. We accomplish this by using a telephone or internet-based randomization system that remotely provides the treatment allocation of patients into a trial. We call this critical concept, “concealment” of allocation.

Whenever possible, it is crucial that the subject themselves are blinded to what treatment they are receiving to eliminate the bias of the placebo effect.^4^ The placebo effect can be observed when a subject knows the treatment they receive, believing that it will benefit them and consequently show improvement, even if the treatment has no effect. This can lead subjects to exaggerate their responses in follow-up, thus skewing results.

The study design should ensure that the two groups in the trial have similar prognostic factors.^4^,^5^ Prognostic factors include age of the subject, stage in the disease process and subject comorbidities. If the experimental group was significantly sicker than the control group, the groups cannot fairly be compared at the end of the study.^4^,^5^ To eliminate bias, stratification can be utilized. Stratification is often performed by a computer-generated randomization system and it ensures randomization is most effective. Stratification ensures that any prognostic factor that may influence outcomes are recognized and accounted for in the initial randomization.^4^ Consequently, the groups in the trial will be similar.

Another issue to consider is whether the subject was analyzed in the group to which they were originally randomized, regardless of whether they receive the intended treatment or not.^4^,^5^ This term, referred to as *intention to treat analysis*, preserves randomization and the balance of prognosis between groups.

A significant insult to a study’s validity is a loss rate of 10% or more of the original subject group.^4^ When there are many subjects unaccounted in the results, it is impossible to deduct if their outcome was negative or positive. Thus, this considerably reduces a study’s power.^4^,^5^

There are resources available meant to aide researchers in developing, conducting and reporting of their RCT that when adhered to, improve the validity of the study’s results. Consort 2010 is a paper that provides guidelines for conducting an RCT^1^ and includes a comprehensive checklist of information that must be included, including methods of blinding, sample size and generalizability of the results.^1^

### What are the results of the trial?

At this point in the appraisal process, the trial’s results must be examined. It is important that results are presented in a way that they are easily interpreted.^4^,^5^ Valuable results to note are calculations for absolute risk reduction, relative risk, relative risk reduction and numbers needed to treat, as well as health-related quality of life responses.^4^,^5^ These are valuable measures to demonstrate the significance and size of the treatment effect.

### Are these results applicable to clinical practice?

Even with a flawless design and significant results, one must not assume that the trial is relevant to clinical practice. For most clinical trials, the eligibility inclusion/exclusion criterion is usually very specific.^4^ For example, a study may only enroll male subjects between the ages of 20 and 50. If a surgeon’s patient bears little similarity to the subjects in the study, the results may not be applicable. When interpreting reported results, a clinician must also decide if the benefits of the treatment outweigh the potential risks and costs.^4^,^5^ If the benefits of the treatment are only marginal, they may not justify initiating the treatment on the subject.

## PRACTICAL EXAMPLE

Guo and colleagues conducted a randomized trial in 85 patients comparing closed intramedullary nailing with minimally invasive plate osteosynthesis using a percutaneous locked compression plate in subjects with a distal metaphyseal fracture in a prospective study.^6^ The mean radiation time and operating time were significantly longer in the locked compression plate group (3.0 vs 2.12 minutes, *P*<0.001 and 97.9 vs 81.2 minutes, *P* <0.001, respectively). At 1 year, intramedullary nailing patients had a higher mean pain score, but better function, alignment and total American Orthopaedic Foot and Ankle surgery scores. These differences, however, were not statistically significant. The authors preferred closed intramedullary nailing for subjects with these fractures.

## THE 3-MINUTE APPRAISAL

Applying the checklist criteria from Table 1, we can quickly determine the validity of the study. Neither treatment examined is novel, although one may assume that the surgeons involved in the study have more experience with one procedure than the other. For this reason, the learning curve applies, although this is unaccounted for in the article.

The trial is said to be randomized, although the details of this are vague. No mention of stratification is made and it is unclear if subjects were analyzed in the group they were originally randomized into. All that is said is that there were no exclusions from the study postrandomization. With this study, concealment postsurgery is essentially impossible and there is no mention of blinding anywhere within the study. As a result, it can be presumed that subjects, surgeons and outcome assessors were all aware of group allocation. The study lists stringent inclusion/exclusion criteria (i.e., only certain classes of fractures were eligible) suggesting that the two groups examined in the study were similar in prognostic factors. Of the original 111 subjects initially enrolled in the study, follow-up was only completed by 85, leaving nearly a quarter of the subjects unaccounted the study’s results.

### What are the results of the study?

The study reports no significant difference was found in the fracture union time of subjects in both groups. The only significant finding reported was the shorter operating time and radiation time for those who had received an intramedullary nailing. Consequently, one may make the assumption that the intramedullary nailing is the superior procedure in the treatment of this type of fracture.

### Are the results applicable to clinical practice?

The results of this particular RCT are undoubtedly applicable to clinical practice. However, one potential limitation may be its ability to be generalized to many tibial fractures as the inclusion/exclusion criteria is very detailed, and may not encompass all patients with such fractures. Lack of difference in functional outcomes with either implant and the increased radiation exposure with plates tends to argue in favor of nails.

## SUMMARY

It is wise to not be limited to one study; the results of several related RCTs should be examined when making a decision regarding clinical practice [Table 2]. One must be critical of all studies encountered, never accepting the results at face value making appraisal essential to evidence-based medicine. Table 2 contains a concise checklist of items to be mindful of when critically appraising an RCT.

**Table 2:** The do’s and do not’s of appraising an RCT

Do	Do not
Ensure all pertinent information regarding method of randomization is included.	Accept all results at face value; if the methodology behind the study is not sound, neither are the results.
Consider the unique challenges existing with surgical RCTs regarding concealment and the learning curve and how this was accounted for.	Ignore the merit of randomized surgical trials simply due to the additional challenges they have.
Assume that if critical information regarding concealment and randomization is not included, it is because it did not occur.	Assume that the patients included in the RCT are similar to those encountered in clinical practice.
Examine sample size and follow-up rates to gauge the power of the study.	Assume that the results from one RCT can be generally applied.
Note the homogeny of the control and experimental group.	
Check to see what outcomes were examined and how the significance of the treatment effect.	
Consider whether the findings of the study can be generalized to clinical practice.	
Examine the evidence from several similar RCTs, conducted independently.	

RCT - Randomized controlled trial
